# Parkinson’s family dynamics and caregiver sense of coherence: A family‐systems approach to coping in Mexico and the United States

**DOI:** 10.1002/agm2.12130

**Published:** 2020-10-26

**Authors:** Teresita Villaseñor, Paul B. Perrin, Emily K. Donovan, Grace B. McKee, Richard S. Henry, Joseph M. Dzierzewski, Sarah K. Lageman

**Affiliations:** ^1^ Hospital Civil Fray Antonio Alcalde Guadalajara Jalisco Mexico; ^2^ Neurosciences Department University of Guadalajara Guadalajara Jalisco Mexico; ^3^ Department of Psychology Virginia Commonwealth University Richmond Virginia United States; ^4^ Department of Physical Medicine and Rehabilitation Virginia Commonwealth University Richmond Virginia United States; ^5^ Hunter Holmes McGuire Veterans Affairs Medical Center Mid‐Atlantic Mental Illness Research Education and Clinical Center Central Virginia VA Health Care System Richmond Virginia United States; ^6^ Department of Neurology Parkinson’s & Movement Disorders Center Virginia Commonwealth University Richmond Virginia United States

**Keywords:** coping, cross‐cultural, family dynamics, Parkinson’s caregivers, sense of coherence

## Abstract

**Objective:**

The population of individuals with Parkinson’s disease (PD) is growing in Mexico and the United States, and there is an increasing need for family members to provide caregiving. This study examined the connections between family dynamics and coping, or sense of coherence, among PD caregivers in Mexico (n = 148) and the United States (n = 105).

**Methods:**

Caregivers completed measures of family dynamics and sense of coherence across indices of comprehensibility, manageability, and meaningfulness.

**Results:**

Although caregivers in Mexico and the United States had similar levels of sense of coherence and family dynamics reflecting strengths/adaptability and being overwhelmed with difficulties, caregivers in Mexico had worse disrupted communication. Family dynamics explained: 24.2% of the variance in caregiver comprehensibility in the United States and 17.5% in Mexico; 34.1% in manageability in the United States and 23.5% in Mexico; and 22.6% in meaningfulness in the United States and 22.7% in Mexico (all *P*s < 0.001). In both Mexico and the United States, family strengths/adaptability uniquely predicted caregiver comprehensibility, manageability, and meaningfulness. Being overwhelmed with difficulties uniquely predicted comprehensibility in Mexico and manageability and meaningfulness in the United States.

**Conclusion:**

The development of family‐systems interventions for PD caregivers to improve family strengths/adaptability and help families deal with difficulties may increase caregiver coping.

## Graphical abstract

This study examined the connections between family dynamics and coping, or sense of coherence, among Parkinson's disease caregivers in Mexico and the United States.

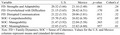

## 1 INTRODUCTION

Parkinson’s disease (PD) is a neurodegenerative disease characterized by motor symptoms, including tremors, limb rigidity, bradykinesia (ie, slow movement), and, later in the course of the disease, postural instability (ie, trouble balancing and falls).^1^, ^2^ Individuals with PD also experience a variety of non‐motor symptoms, including cognitive, neuropsychiatric, sleep, autonomic, and olfactory dysfunction and disturbances.^1^ There are over half a million individuals with PD in the United States,^3^ and around 125 000 in Mexico.^4^ Though PD is a disease of unknown etiology, both environmental and genetic factors are believed to play a role in its development.^5^ Diagnosis of PD is complicated, as it is diagnosed based on clinical symptom clusters.^6^ Therefore, individuals with PD and their caregivers may be left to manage PD‐related non‐motor symptoms (eg, mood, sleep, and gastrointestinal issues) alone, until the cardinal motor symptoms are present, leading to initiation of medical care. After diagnosis and beyond, the progressive nature of the disease requires caregivers to provide increasing care over time.

PD caregiving duties include providing instrumental support (eg, helping with dressing and bathing), emotional support, and informational support (eg, coordinating care and managing medications).^7^ This care can be time‐consuming and, as a result, PD caregivers may not be able to spend as much time with friends and family, resulting in social isolation.^8^ Compared to the general population, PD caregivers have a significantly lower quality of life and mental health,^9^, ^10^, ^11^ with spousal PD caregivers experiencing more episodes of chronic illness than non‐caregiver spouses^8^ and PD caregivers experiencing poor‐quality sleep.^7^ Numerous studies have documented negative psychological outcomes, such as anxiety and depression, as being directly associated with perceived burden of PD caregivers.^7^, ^10^, ^12^, ^13^, ^14^, ^15^, ^16^, ^17^ While maintenance of caregiving responsibilities over time can lead directly to negative outcomes for PD caregivers, stress associated with caregiving can be present even at the early stages of the disease.^18^ Additionally, PD caregivers may face stigma due to feelings of shame and pity surrounding the disease and its symptoms.^19^ Caregivers may ultimately end up feeling burned out when their caregiving duties go beyond their resources, and this could lead to institutionalization of the individual with PD.^7^, ^20^ Therefore, it is important to identify caregiver strengths and factors influencing coping ability, so that caregivers can continue to fulfill their critical role.

Given the impact of caregiving on caregivers of individuals with PD, researchers have begun trying to identify factors that may serve as buffers for negative mental and physical health outcomes for PD caregivers. In a study of PD caregivers in Spain, coping responses were predictive of caregivers’ and care recipients’ psychological adjustment as well as quality of life of caregivers.^21^ In addition to coping styles or responses, there is a body of literature evaluating an individual’s outlook and self‐efficacy regarding comprehensibility, manageability, and meaningfulness of stressful life events, also known as sense of coherence (SOC).^22^ From an SOC standpoint, comprehensibility is the feeling that one’s world and environment make sense or are consistent; manageability is the feeling that there are adequate resources to meet demands; and meaningfulness is the feeling that the demands are worth the time and effort.^23^, ^24^ In PD caregivers, a low SOC has been found to be one of the most important predictors of caregiver burden.^13^ Similarly, in caregivers of individuals with dementia, caregivers’ lower SOC predicted higher anxiety, depression, and burden.^25^ SOC is also a strong predictor of health‐related quality of life in a general sample of informal caregivers.^26^ When considering the association of SOC with vital caregiver outcomes, it is important to consider how SOC may develop and interact in different ways cross‐culturally.

Qualitative research suggests that, while PD caregivers face many challenges, family support is an important factor for the caregiving experience.^27^ Since most PD caregivers are spouses or other family members, family dynamics can predict or be a buffer for important caregiver outcomes. Family dynamics describe how families conduct their lives and relationships.^28^ Aspects of family dynamics that are considered particularly important for caregiver outcomes are strengths and adaptability within the family, the sense of feeling overwhelmed by difficulties, and disrupted communication.^28^ Previous studies have shown that family dynamics are a good predictor of caregiver mental health and strengths in caregivers of individuals with other neurological conditions. For example, family dynamics were associated with both resilience and SOC in Argentinian dementia caregivers, with family problems predicting both resilience and SOC, empathy predicting resilience, and communication predicting SOC.^29^ In other groups of caregivers of individuals with neurological conditions (ie, dementia or traumatic brain injury) across Latin America, family dynamics have predicted caregiver depression,^30^, ^31^ stress,^31^ satisfaction with life,^31^, ^32^ and burden.^31^, ^32^, ^33^ In general, healthier family dynamics are associated with stronger caregiver mental health as well as care‐recipient mental health; however, one study found that family dynamics predicted many more caregiver mental health outcomes than care‐recipient mental health outcomes.^30^ Less is known about family dynamics among familial caregivers of individuals with PD.

Most work evaluating the mental and physical health outcomes of PD caregivers has focused on populations of caregivers in the United States and Western Europe. However, less is known about PD caregivers in Mexico and the cross‐cultural differences that may impact the caregiving role and coping ability for those caregivers. There are several cultural values that may influence how PD caregivers approach and internalize their caregiving role.^34^ For instance, *familismo* is a value within Latin American cultures that encompasses an emphasis on respecting elders,^35^ relying on other family members, and being obligated to others within the family.^36^ Due to *familismo*, a PD caregiver in a Latin American family may feel stress or guilt associated with not meeting the expectations of their family.^37^ On the other hand, *familismo* could allow a PD caregiver within a Latin American family to perceive their role as less burdensome than someone without that cultural value.^38^ In the same vein, a study of college‐aged adults demonstrated that family functioning (ie, family dynamics) did not directly predict willingness to care for a family member; however, family dynamics indirectly predicted willingness to care through family values.^39^ Therefore, cultural family values may contribute to the relationship between family dynamics and caregiver outcomes for PD caregivers.

Twice as many individuals will be living with PD in 2030 as there were in 2005,^40^ resulting in an increasing need to examine cultural differences in the caregiving role to facilitate understanding and support for the diverse, growing number of PD caregivers. Given that healthy family dynamics have been shown to be an important element of coping in diverse samples of caregivers, the purpose of the present study was to examine the connections between family dynamics and SOC, or coping ability, of PD caregivers differentially in the United States and Mexico. The study is also the first, to our knowledge, to study these constructs in PD caregivers and cross‐culturally.

## 2 METHODS

### 2.1 Participants

At specialty PD clinics at major public universities in both the United States and Mexico, informal caregivers of individuals with PD (N = 253) were recruited for the current study. To be eligible to participate, individuals needed to be (a) the primary caregiver of an individual with a physician’s diagnosis of PD who had been seen at one of the clinics, (b) fluent in either Spanish (for the Mexican site) or English (for the US site), and (c) at or over the age of 18 years. For further information about the sample demographics, see Table 1.

**Table 1:** Participant demographics

Variables	United States (n = 105)	Mexico (n = 148)
Caregiver		
Age, years, mean (SD)	68.73 (8.36)	53.66 (14.96)
Hours of care/week, mean (SD)	59.38 (64.56)	107.39 (61.34)
Months as a caregiver, mean (SD)	46.78 (81.33)	52.38 (49.22)
Sex, %		
Male	31.4	23.6
Female	68.6	76.4
Race/ethnicity, %		
Latino/Hispanic	—	100.0
White/European (non‐Latino)	92.4	—
Asian/Asian American/Pacific Islander	2.9	—
Black/African American (non‐Latino)	2.9	—
Multi‐racial/multi‐ethnic	1.0	—
Other	1.0	—
Social class, %		
Upper	2.9	0.7
Upper‐middle	63.8	22.3
Lower‐middle	23.8	37.2
Working	9.5	24.3
Lower	—	15.5
Highest completed education level, %		
Doctorate degree	7.6	—
Master’s degree	21.9	2.0
4‐year college degree	33.3	16.2
2‐year technical college degree	11.4	13.5
High school/GED	25.7	5.4
Grade school	—	58.1
No formal education	—	4.7
Care recipient		
Age, years, mean (SD)	71.61 (8.13)	65.68 (10.78)
Months since PD diagnosis, mean (SD)	92.25 (82.84)	63.22 (60.88)
Sex, %		
Male	64.8	52.0
Female	35.2	48.0

### 2.2 Procedure

Following protocol approval from both institutions’ institutional review boards, caregivers from PD clinics in Mexico and the United States were recruited through various means, including: direct contact, phone, email, flyers, and word of mouth. Informal caregivers who accompanied patients to medical appointments at the clinics were also provided information about the study. After providing informed consent, caregivers completed self‐report questionnaires in the clinics (often while the individual with PD was being seen for appointments) assessing family dynamics, caregiver SOC, and demographic information.

### 2.3 Measures

#### 2.3.1 Family dynamics

Family dynamics were assessed using the SCORE‐15.^28^ This 15‐item self‐report measure has three subscales: Strengths and Adaptability, Overwhelmed by Difficulties, and Disrupted Communication. The reliability of the Strengths and Adaptability subscale for the current study was α = 0.83 in the United States and α = 0.70 in Mexico, that for the Overwhelmed by Difficulties subscale was α = 0.79 in the United States and α = 0.78 in Mexico, and that for the Disrupted Communication subscale was α = 0.73 in the United States and α = 0.64 in Mexico. As there is no Spanish version of this measure available, the current study used the Chapman and Carter^41^ translation method, in which a bicultural, bilingual researcher translates the measure into Spanish and a different bicultural, bilingual researcher back translates the measure into English. Using this procedure, any discrepancies between researchers were addressed and resolved. In the current study, the Overwhelmed with Difficulty and Disrupted Communication subscales were reflected so that on all subscales, higher scores suggest healthier family dynamics.

#### 2.3.2 Caregiver SOC

Caregiver SOC was measured using the Sense of Coherence (SOC‐13) scale.^23^ This 13‐item self‐report measure uses 7‐point Likert‐type response options relevant to each question and has three subscales: Comprehensibility, Manageability, and Meaning. A validated Spanish version was used in the current study.^42^ The reliability of the Comprehensibility subscale for this study was α = 0.71 in the United States and α = 0.67 in Mexico, that for the Manageability subscale was α = 0.61 in the United States and α = 0.73 in Mexico, and that for the Meaning subscale was α = 0.65 in the United States and α = 0.65 in Mexico. Higher scores on each of the subscales reflect greater SOC.

## 3 RESULTS

### 3.1 Correlation matrix

A correlation matrix was generated to examine the bivariate relationships among the three types of family dynamics and the three types of caregiver SOC, differentially by site (Table 2). The correlation matrix suggested that family dynamics and caregiver SOC were strongly positively correlated at both sites, and all family dynamics were positively correlated with all forms of SOC, without noticeable magnitude differences by site.

**Table 2:** Correlations between family dynamics and caregiver sense of coherence by site

Variable	1	2	3	4	5	6
1. FD: Strengths/adaptability	—	0.500	0.542	0.346	0.448	0.440
2. FD: Overwhelmed with difficulty	0.590	—	0.661	0.373	0.384	0.378
3. FD: Disrupted communication	0.579	0.632	—	0.322	0.346	0.340
4. SOC: Comprehensibility	0.461	0.412	0.319	—	0.656	0.565
5. SOC: Manageability	0.539	0.494	0.349	0.758	—	0.449
6. SOC: Meaningfulness	0.426	0.411	0.266	0.586	0.541	—

Abbreviations: FD, family dynamics; SOC, sense of coherence.

Correlations below the diagonal are from the United States and those above the diagonal are from Mexico. All correlation coefficients were statistically significant at *P* < 0.01.

### 3.2 Site comparisons

A series of analyses of variance (ANOVAs) compared overall levels of family dynamics and caregiver SOC by site (Table 3). These comparisons found that caregivers in the United States had higher scores on the reflected Disrupted Communication subscale of the SCORE‐15 than caregivers in Mexico, suggesting healthier communication in families of US caregivers. Caregivers at the two sites had comparable scores on the Strengths and Adaptability subscale, as well as on the Overwhelmed by Difficulties subscale. Caregivers also reported similar levels of SOC across all three subscales.

**Table 3:** Site comparisons

Variable	US	Mexico	*P* value	Cohen’s *d*
FD: Strengths and adaptability	20.32 (3.66)	21.16 (3.48)	0.063	0.24
FD: Overwhelmed with difficulties	21.15 (3.65)	20.42 (4.51)	0.170	0.18
FD: Disrupted communication	21.22 (3.33)	20.06 (3.81)	0.013	0.32
SOC: Comprehensibility	25.70 (5.42)	26.02 (6.38)	0.672	0.05
SOC: Manageability	21.47 (3.92)	22.04 (5.58)	0.365	0.12
SOC: Meaningfulness	22.85 (3.67)	22.82 (4.80)	0.957	0.01

Abbreviations: FD, family dynamics; SOC, sense of coherence.

Values for the US and Mexico columns represent means (standard deviations).

### 3.3 Regressions

A series of simultaneous multiple regressions were run differentially by site in which the predictor variables were the three types of family dynamics and the criterion variables were the three types of caregiver SOC. In the United States, family dynamics explained 24.2% of the variance in caregiver comprehensibility, *F*(3, 104) = 10.76, *P* < 0.001. Within this regression, strengths and adaptability (β = 0.34, *P* = 0.004) was a statistically significant unique predictor. All other predictors were not statistically significant (all *P*s ≥ 0.066).

In Mexico, family dynamics explained 17.5% of the variance in caregiver comprehensibility, *F*(3, 147) = 10.17, *P* < 0.001. Within this regression, both strengths and adaptability (β = 0.20, *P *= 0.035) and feeling overwhelmed with difficulties (β = 0.24, *P* = 0.025) were statistically significant unique predictors. Disrupted communication was not statistically significant (*P* = 0.572).

In the United States, family dynamics explained 34.1% of the variance in caregiver manageability, *F*(3, 104) = 17.40, *P* < 0.001. Within this regression, strengths and adaptability (β = 0.40, *P* < 0.001) and feeling overwhelmed with difficulties (β = 0.30, *P* = 0.008) were statistically significant unique predictors. Disrupted communication was not statistically significant (*P* = 0.492).

In Mexico, family dynamics explained 23.5% of the variance in caregiver manageability, *F*(3, 147) = 14.78, *P* < 0.001. Within this regression, the level of strengths and adaptability (β = 0.33, *P* < 0.001) was a statistically significant unique predictor. All other predictors were not statistically significant (all *P*s ≥ 0.055).

In the United States, family dynamics explained 22.6% of the variance in caregiver meaningfulness, *F*(3, 104) = 9.83, *P* < 0.001. Within this regression, strengths and adaptability (β = 0.31, *P* = 0.008) and feeling overwhelmed with difficulties (β = 0.29, *P* = 0.019) were statistically significant unique predictors. Disrupted communication was not statistically significant (*P* = 0.422).

In Mexico, family dynamics explained 22.7% of the variance in caregiver meaningfulness, *F*(3, 147) = 14.13, *P* < 0.001. Within this regression, the level of strengths and adaptability (β = 0.32, *P* < 0.001) was a statistically significant unique predictor. All other predictors were not statistically significant (all *P*s ≥ 0.060).

## 4 DISCUSSION

The purpose of this study was to examine the connections between family dynamics and coping ability, or SOC, among PD caregivers in Mexico and the United States. Although caregivers in Mexico and the United States had similar levels of SOC and family dynamics reflecting both strengths/adaptability and being overwhelmed with difficulties, caregivers in Mexico had worse disrupted communication. Family dynamics explained: 24.2% of the variance in caregiver comprehensibility in the United States and 17.5% in Mexico; 34.1% of the variance in caregiver manageability in the United States and 23.5% in Mexico; and 22.6% of the variance in caregiver meaningfulness in the United States and 22.7% in Mexico. In both Mexico and the United States, family strengths/adaptability uniquely predicted caregiver comprehensibility, manageability, and meaningfulness. Being overwhelmed with difficulties uniquely predicted caregiver comprehensibility in Mexico and manageability and meaningfulness in the United States.

Overall, the mean difference comparisons revealed that caregivers at the US and Mexico sites reported similar levels of caregiver comprehensibility, manageability, and meaningfulness (eg, SOC). Caregivers’ SOC has been previously linked with burden,^13^, ^25^ anxiety and depression,^25^ and health‐related quality of life.^26^ These results provide preliminary evidence that SOC develops in similar ways across these cultures; however, even if this finding is replicated in additional research, it could be that SOC interacts with other factors in different ways cross‐culturally and could therefore differentially impact outcomes such as caregiver burden and quality of life.

Interestingly, the results comparing mean levels of family dynamics showed that although caregivers in the United States and Mexico reported similar levels of strengths and adaptability and feeling overwhelmed with difficulties, the two sites showed significant differences in scores on disrupted communication. These results may or may not necessarily suggest that Mexican families have greater disruptions in communication than do US families. Although the participants at the Mexico site completed a version of the SCORE‐15 that was translated into Spanish, it may be that the SCORE‐15 does not fully capture the construct of family communication as it applies to populations outside of the United States and Western Europe. Thus, if the SCORE‐15 was developed using Eurocentric norms around family dynamics and communication, it may inadequately assess this construct in countries with differing norms and values even when the items are translated appropriately. That is, cultural differences in what constitutes healthy family dynamics, including communication, may systematically differ across the two samples, which could result in differential validity of the measure. That could suggest that the estimates of disrupted communication in caregivers from the Mexico site provide an incomplete assessment of their actual family communication dynamics. Indeed, the α value for the Disrupted Communication subscale was particularly low for the Mexico site (α = 0.64), which could suggest that it does not fully capture what appears to be a complex and heterogeneous construct. Further work in this vein is necessary to more fully understand cross‐cultural differences in family dynamics and to ensure adequate assessment of these constructs in non‐English‐speaking samples.

Results from the current study provide evidence that family dynamics play an important role in PD caregiver coping in both the United States and Mexico, with family dynamics variables explaining 17.5% to 34.1% of the variance in caregiver comprehensibility, manageability, and meaningfulness. These findings are consistent with previous work, including US caregivers of individuals with Alzheimer’s^43^ and caregivers of individuals with dementia in Argentina,^29^ but are among the first to demonstrate this association within a Latin American sample of PD caregivers. Thus, despite potential cross‐cultural differences between the two samples (eg, degree of emphasis on values like *familismo*), these results provide consistent evidence that family dynamics are an important factor for PD caregivers in both the United States and Mexico.

Notably, the Strengths and Adaptability subscale was the single largest unique predictor of PD caregiver SOC for both sites. Moreover, it was the only predictor to demonstrate statistical significance across all six models. Although studies have supported the relation between family dynamics and SOC more generally, this particular finding is consistent with previous research showing the importance of coping responses,^21^ adaptability/flexibility,^44^ resilience,^45^ and family support ^27^ in caregiver outcomes. Overall, this finding suggests that strengths and adaptability within the family may be of particular importance to PD caregiver SOC, and additional research could investigate whether the experience of family strengths and adaptability may buffer against additional stressors or negative aspects of caregiving.

Across both sites, being overwhelmed with difficulties was also a unique predictor of caregiver SOC, though of different aspects of SOC. For caregivers at the US site, being overwhelmed with difficulties predicted caregiver manageability and meaningfulness. In contrast, for caregivers at the Mexico site, being overwhelmed with difficulties predicted caregiver comprehensibility alone. Although these results underscore the importance of family dynamics in caregiver SOC more generally, the site differences may reflect cross‐cultural differences in the caregiving experience. For example, in Mexico, PD caregivers may receive substantial emotional support from extended family members and communities, as reflected in the value of *familismo*,^36^ when difficulties are perceived as overwhelming; this experience may run counter to cultural norms around caregiving (eg, to such an extent that one’s worldview and environment no longer feel consistent^34^). However, in the United States, in which individualism may be a more prevalent cultural norm, a sense of overwhelming difficulties may relate more directly to caregivers’ perception that their resources are inadequate and that demands of caregiving may not be worth the time and effort.

## 5 CLINICAL IMPLICATIONS

Results from the current study suggest that family dynamics play an important role in caregiver SOC. Family strengths/adaptability and the perception of being overwhelmed with difficulties represent clear targets for interventions aiming to improve caregiver mental health and quality of life. Through this pathway, interventions may also indirectly improve the quality of care that caregivers are able to provide to individuals with PD. Future clinical intervention research could therefore focus on adapting or developing family‐systems interventions for families affected by PD. Further, these interventions would benefit from taking into account the cross‐cultural context in order to adapt to the needs of non‐US families. For example, a recent pilot study was conducted examining the results of a brief family‐based intervention for individuals with spinal cord injury and their families in Colombia, South America. The eight‐session intervention addressed skills such as making meaning, boundary making and other healthy family dynamics, and communicating effectively. Results showed significant improvements on measures of mental health, burden, and perceived problem‐solving skills.^46^ Interventions with similar skills targets could therefore be adapted for use with families of individuals with PD.

### 5.1 Limitations and future directions

Although this study is among the first to investigate associations between family dynamics and caregiver SOC in the United States and Mexico, these findings should take into account several important limitations. First, sample characteristics may limit the generalizability of our findings. Most caregivers in the US sample (92.4%) identified as White/European (non‐Latino) and had higher education levels and socioeconomic statuses than the general US population, with 29.5% attaining graduate degrees and two‐thirds falling within the upper‐middle to upper classes. In contrast, only 2% of the caregivers in Mexico reported attaining a graduate degree, and a majority reported attending grade school only (58.1%) or receiving no formal education (4.7%). Future studies would benefit from recruiting US participants demonstrating greater diversity in race/ethnicity, socioeconomic status/income, and education levels that are more representative of the general US population in order to facilitate more accurate cross‐cultural comparisons. Further, because the current study only collected data from one city in Mexico, this limits generalizability to other areas of Mexico and other Latin American countries. Future research would therefore also benefit from collecting data from multiple sites in both the United States and Mexico in order to improve generalizability; for example, researchers are encouraged to consider including rural sites in addition to urban/suburban sites.

Because of the cross‐sectional nature of the study, causality cannot be inferred from these results. Although the current study conceptualized family dynamics as contributing to caregiver SOC, caregivers with low self‐efficacy regarding comprehensibility, manageability, and meaningfulness of stressful life events may also be less adaptable, less likely to enact effective communication skills, and more likely to perceive difficulties as overwhelming. Researchers interested in investigating the causal nature of these associations could utilize longitudinal cross‐lagged panel designs in order to more appropriately measure and test for causal links between family dynamics and SOC. Lastly, as discussed above, the measure of family dynamics used in the current study (SCORE‐15) has not been validated for use in Spanish‐speaking populations and may not fully capture cultural norms in communication and other aspects of healthy family relationships. Future research would benefit from more closely investigating cross‐cultural differences in family dynamics and their differential impact on caregiver mental and physical health, as well as on broader family outcomes.

## 6 CONCLUSION

The current study investigated the associations between aspects of family dynamics and caregiver SOC in samples of PD caregivers in the United States and Mexico. Caregivers reported similar levels of all three aspects of SOC, as well as aspects of family dynamics reflecting strengths/adaptability and being overwhelmed with difficulties; caregivers in Mexico reported greater levels of disrupted communication than did caregivers in the United States. Strengths and adaptability explained significant amounts of variance in caregiver comprehensibility, manageability, and meaningfulness across both sites. Being overwhelmed with difficulties significantly predicted comprehensibility for caregivers in Mexico and manageability and meaningfulness for caregivers in the United States. Differences in the association between being overwhelmed with difficulties and aspects of SOC may relate to differences in cultural norms around caregiving in the United States and Mexico. Interventions targeting strengths/adaptability and problem management in individuals with PD and their caregivers may benefit from adapting family‐based interventions that have already been developed for individuals with spinal cord injury and their families or other similar populations. Culturally tailored family‐based interventions may impact caregiver well‐being by targeting aspects of family dynamics and could therefore indirectly improve the quality of care that individuals with PD receive.

## CONFLICTS OF INTEREST

The authors report no conflicts of interest.

## AUTHOR CONTRIBUTIONS

T.V., P.B.P., and S.K.L. were involved in study design and collected the data. P.B.P., E.K.D., G.B.M., and R.S.H. drafted the manuscript. P.B.P. and R.S.H. statistically analyzed. T.V., P.B.P., G.B.M., J.M.D., and S.K.L. edited the manuscript.

## Data Availability

The data used to support the findings of this study are available from the corresponding author upon request.
